# Creation and Implementation of a Large-Scale Geriatric Interprofessional Education Experience

**DOI:** 10.1155/2020/3175403

**Published:** 2020-07-25

**Authors:** Colleen Mcquown, Rami A. Ahmed, Patrick G. Hughes, Fabiana Ortiz-Figueroa, Jennifer C. Drost, Diane K. Brown, Sue Fosnight, Susan Hazelett

**Affiliations:** ^1^Louis Stokes Cleveland Veterans Affairs Medical Center, Cleveland, OH, USA; ^2^Department of Emergency Medicine, Indiana University School of Medicine, Indianapolis, IN, USA; ^3^Charles E. Schmidt College of Medicine, Florida Atlantic University, Boca Raton, FL, USA; ^4^Department of Emergency Medicine, University of Texas Health Science Center at San Antonio, San Antonio, TX, USA; ^5^Department of Geriatrics, Summa Health System, Akron, OH, USA; ^6^College of Health Professions, University of Akron, Akron, OH, USA

## Abstract

The care of the older adult requires an interprofessional approach to solve complex medical and social problems, but this approach is difficult to teach in our educational silos. We developed an interprofessional educational session in response to national requests for innovative practice models that use collaborative interprofessional teams. We chose geriatric fall prevention as our area of focus as our development of the educational session coincided with the development of an interprofessional Fall Risk Reduction Clinic. Our aim of this study was to evaluate the number and type of students who attended a pilot and 10 subsequent educational sessions. We also documented the changes that occurred due to a Plan-Do-Study-Act (PDSA) rapid-cycle improvement model to modify our educational session. The educational session evolved into an online presession self-study didactic and in-person educational session with a poster/skill section, an interprofessional team simulation, and simulated patient experience. The simulated patient experience included an interprofessional fall evaluation, team meeting, and presentation to an expert panel. The pilot session had 83 students from the three sponsoring institutions (hospital system, university, and medical university). Students were from undergraduate nursing, nurse practitioner graduate program, pharmacy, medicine, social work, physical therapy, nutrition, and pastoral care. Since the pilot, 719 students have participated in various manifestations of the online didactic plus in-person training sessions. Ten separate educational sessions have been given at three different institutions. Survey data with demographic information were available on 524 participants. Students came from ten different schools and represented thirteen different health care disciplines. A large-scale interprofessional educational session is possible with rapid-cycle improvement, inclusion of educators from a variety of learning institutions, and flexibility with curriculum to accommodate learners in various stages of training.

## 1. Introduction

The Institute of Medicine (IOM) report from the Committee on the Future Health Care Workforce for Older Americans says that all licensure of medical professionals should require documentation on the competence in the care for older adults [1]. Geriatric medical educators have recognized the need to emphasize multidisciplinary team care as a core competency [2]. Over the last 16 years, multiple studies have highlighted the benefits of interprofessional education (IPE) to improve knowledge and skills needed for collaborative behavior at all levels of training in the health care field [3–7]. Safety experts advise IPE as a method to reduce adverse medical events [3, 8–10]. National and international trends in health care education reflect the desire to provide students with IPE [9–13].

The IOM suggests simulation be used in IPE to train novices and practitioners who would normally work together [14]. Team training using simulation is a proven method to teach both clinical and nonclinical skills [4, 5, 15, 16]. Despite a wealth of knowledge supporting the benefits of IPE, many studies also highlight lessons learned with implementation [17, 18]. Scheduling students from different disciplines and educational paths seems to be a universal problem limiting longitudinal programs [9, 11, 13, 17, 18]. Students also prefer hands-on training to observation or online discussions [17, 19, 20]. Students typically report enhanced realism and immediate feedback provided from standardized patients in medical simulation in comparison to more traditional methodologies [19]. This is especially true when students are learning the effects of their decision-making as a collective group in IPE sessions [20]. Students reported that interprofessional team training made them more prepared to work in such teams in real life and appreciate working with students from other disciplines [17, 20]. Lack of faculty development or need for IPE-specific faculty development has been cited as a challenge for IPE implementation and success [6, 10, 13, 21, 22].

We sought to develop an IPE curriculum to train undergraduate, graduate, and specialized health service providers with a focus on geriatric fall prevention. We chose to focus on geriatric fall prevention for two reasons. First, it was done in conjunction with the development of an IP (interprofessional) Fall Risk Reduction Clinic (FRRC) that included clinician educators who could work as role models, facilitators, and advisors. Second, there were local needs of students requiring more geriatric focused education. Fall prevention is an ideal topic to tackle with an IP team as the causes and solutions are multifactorial and involve social, medical, and community resources [23].

Wagner's model of chronic care framework can be used to model IP clinics and IPE events. The Wagner model suggests that patients with chronic complicated conditions require effective communication across health care disciplines to assure optimal health [24, 25]. Studies showed that students like simulation experiences because they reflect real-life scenarios [17, 26].

We present here our framework for the development of the IPE curriculum and the evolution of the curriculum via a continuous process improvement model. The original plan was for all students to experience IP teamwork by attending the educational session and the FRRC. However, given the rapid expansion requested by participating institutions and limited patient volume at the FRRC, an IP simulation was created to fill the gap. The resulting IPE session was done in two parts and included an online presession and a half-day in-person session. The aim of the study was to evaluate the type and number of students that the program accommodated, as well as to document the changes that occurred due to the Plan-Do-Study-Act model.

## 2. Methods

### 2.1. Project Overview

The NEPQR-ICPC program through HRSA funds projects that promote nursing educational opportunities in team-based, interprofessional clinical practice and disseminating collaborative practice models. Our funded project grant had two aims: (1) to establish a nurse-led IP Fall Risk Reduction Clinic at the sponsoring hospital and (2) to create an educational experience that would increase the number of health professions students prepared to work in IP practice environments. Students participated by working with clinicians as part of the FRRC and by attending the IP educational session. The program's original aim was to train nursing, medical, pharmacy, physical therapy (PT), and paramedic students. After the pilot and discussion with the NEPQR-ICPC program office, we were able to expand the educational experience to accommodate hundreds of students.

We used TeamSTEPPS® from the Agency for Healthcare Research and Quality (AHRQ), division of the US Department of Health and Human Services, as the teamwork curriculum [27]. TeamSTEPPS® is an evidence-based set of teamwork tools, curriculum, webinars, and in-person training to improve communication and patient safety [27]. Facilitators were given the opportunity to participate in TeamSTEPPS® training and subsequently Master Training. TeamSTEPPS® concepts were also incorporated into the educational session [28].

### 2.2. Setting

The sponsoring hospital that houses the FRRC is a large, urban tertiary care center, community hospital with a medical school affiliation. The hospital also sponsors a state-accredited emergency medical services (EMS) school with paramedic and emergency medical technician (EMT) certification courses and a clinical pastoral care program. Collaborators also include a large, urban university system with a College of Health Professions which includes nursing, nutrition, exercise science, social work, counseling, and speech therapy.

Grant members created the FRRC which started seeing patients in the fall of 2015. The clinic was staffed with a geriatric nurse practitioner or geriatrician, nurse, pharmacist, and physical therapist who performed the patient evaluation. Patients who had fallen or had been identified at high risk for falling were referred to the clinic by emergency, inpatient, and outpatient health care providers or the area agency on aging. Criteria for referral were independently chosen by each referring provider. The FRRC was listed as part of the geriatric outpatient services available on the hospital website, so patients could also self-refer. FRRC geriatrician and pharmacist worked with hospital discharge coordinators to refer patients who had fallen in the hospital or were hospitalized as a result of a fall. Clinic was a half-day one-two times a week, and each patient was scheduled for an hour appointment. Patients were presented to a full IP team that met twice a week and included the nurse practitioner, nurse, geriatrician, pharmacist, physical therapist, emergency medicine physician, primary care physician, social worker, paramedic, and home health representative. The team would create a fall prevention plan for the patient that included medication recommendations, home modifications, outpatient referrals, and community resources. A team member would then follow up with the patient and his or her primary care doctor.

### 2.3. Participants

#### 2.3.1. Grant Team and Facilitators

The grant team members represented nursing, medicine (geriatrics, emergency medicine, and primary care), pharmacy, EMS, and physical therapy. Grant team members participated in one or both portions of the grant—the FRRC and the IP educational activity. Grant team members acted as facilitators for the educational sessions. In addition, educators from the students' programs were invited to participate as facilitators. These facilitators were given specific roles within the educational session by the grant staff.

#### 2.3.2. Students

Students participated in the FRRC activities and the IPE session. Students who participated in the FRRC were precepted by grant members. Grant members recruited clinical instructors, course directors, and preceptors to facilitate student participation in the IPE session. The instructors/directors/preceptors were given an overview of the educational activity. It was up to these individuals to decide the nature of their students' participation, whether it was mandatory, optional, or in lieu of other training. Some grant members who were also clinical instructors or preceptors also recruited their own students. Students came from the hospital, medical university, community EMS training program, and the local universities (Table 1). Students could be required to participate in the IPE session by their instructor/director/preceptor, but participation in all evaluations of the activity was voluntary. IPE session students were a mixture of community, undergraduate, and graduate programs, but all had declared a major or focus within a clinical health care profession, as they were identified by their clinical instructors.

### 2.4. Evaluation

Student participants were given opportunities for feedback during the educational sessions via open discussion and by anonymous open answer paper surveys. During the pilot session, students were given paper attitude and knowledge surveys. Facilitators (both grant and invited) also completed written evaluations of the educational activity at the conclusion of each session.

Students were asked to participate anonymously in a pre-post survey on IP core competencies using the Interprofessional Socialization and Valuing Scale (ISVS) [29]. ISVS is a validated tool used to assess behaviors, beliefs, and attitudes about interprofessional practice [29, 30]. This was offered when the students signed in to complete their online portion of the educational session. Completing the ISVS was voluntary and not required for participation in the educational session.

The hospital Institutional Review Board (IRB) reviewed the grant and planned methods of evaluation and determined it to be program evaluation rather than research and thus exempted from review, except for the portion evaluating students on interprofessional core competencies. Reciprocity for IRB approval was obtained from partnering institutions.

### 2.5. Framework for IP Education Session Development

#### 2.5.1. Design

The design of the project was a Plan-Do-Study-Act (PDSA) model [31]. For the educational portion, an implementation plan was decided a priori and included three phases of implementation: pilot, modification, and large-scale implementation. In addition to the 3 phases of the roll out, team members met at least monthly during the modification and the implementation phases as part of the PDSA model for rapid cycle improvements and adjustments. There were separate PDSA meetings for the FRRC, but the evolution of the clinic is not the scope of this paper.

### 2.6. Phase 1: Pilot Educational Session and Fall Risk Reduction Clinic Startup

The clinic and the educational session planning meetings occurred simultaneously in the summer and fall of 2015. The initial educational session was planned as a pilot at the hospital's simulation center. The plan with the development of the FRRC was to have the students who attended the educational session see patients either in the clinic or during the follow-up home visits and participate in IP team meetings on the patients they saw.

The pilot educational session consisted of an online didactic that was to be completed prior to the in-person educational session. The live educational session included skills stations and a simulation case. It was conducted in the sponsoring hospital's simulation lab. The session was held on one afternoon and all students participated simultaneously. The skills stations included posters and instruction for hands-on application of knowledge from the online didactics on the different aspects of geriatric fall risks and prevention such as mobility, medication, home safety, nutrition, and cognition. The simulation case was unique in that it was not a simulation of a patient encounter, but a simulation of an IP team meeting. The next step of the educational intervention was for the students to participate in patient care with the FRRC. During the pilot, students were given the ISVS before and after online didactic and after simulation session. Completing the survey was voluntary.

For the IP team meeting simulation session, the students began in separate professional huddles. In the huddle, profession-specific details were disclosed in a patient report/hand-off style. A real case with patient identifiers removed was provided by the hospital geriatric team and used by the facilitators to create a patient narrative. The students were then divided into IP teams. Each IP team consisted of 8–10 students representing different professions. In the IP teams, students presented the assessment findings gathered during the huddles. Students were tasked with developing an evidence-based plan of care based on profession-specific assessments and effective team communication. Facilitators were present to guide students through the process, model effective communication, or stimulate discussion as needed. Facilitators led post-simulation debriefings immediately after the simulation to reflect on the team's priorities of care, team communication, and decision-making.

### 2.7. Phase 2: Reflection and Modification

Multiple planned sessions of the clinical and educational team reviewed the pilot experience and the educational experience available to the students in the clinic. Grant team members identified barriers and challenges with brainstorming sessions on solutions (Table 2). This time was also intended to review the curriculum and the students' overall educational experience. Team members were also given educational opportunities as lack of faculty development has been cited as a challenge for IPE implementation and success [6, 10, 13, 21, 22]. Lectures were given on motivational interviewing, nutrition, and emotional intelligence. Formal debriefing training was offered through the medical university. Grant team members could also do in-person or self-paced TeamSTEPPS® training through AHRQ, division of the US Department of Health and Human Services [27, 28].

The success of the pilot program prompted professional educators from different disciplines and institutions to participate in the modification and implementation of the educational sessions. University-based educators in speech therapy, exercise science, occupational therapy, nutrition, chaplaincy, nursing, and social work were recruited for the educational session in addition to the core group listed on the grant. The response from the local health care educational community was overwhelmingly positive, and requests for additional sessions were considered. The schools expressed a need for more IP training opportunities for their students, and our program had the unique ability to modify the sessions to meet the needs of the hosting institutions. As a result of the increased demand and need for flexibility to accommodate student needs, team members looked outside the sponsoring hospital to hold additional training sessions. Requests for IP experience came from multiple affiliated, but not originally intended, local universities and colleges. Hosting institutions did not limit the session to only their students, so students from all of the sponsoring institutions were able to participate. Students were assigned to participate by the clinical preceptor, classroom instructor, or professor as part of their school assignments, or simply informed by their teacher of the voluntary opportunity as a supplement to their education.

**Figure 1:**
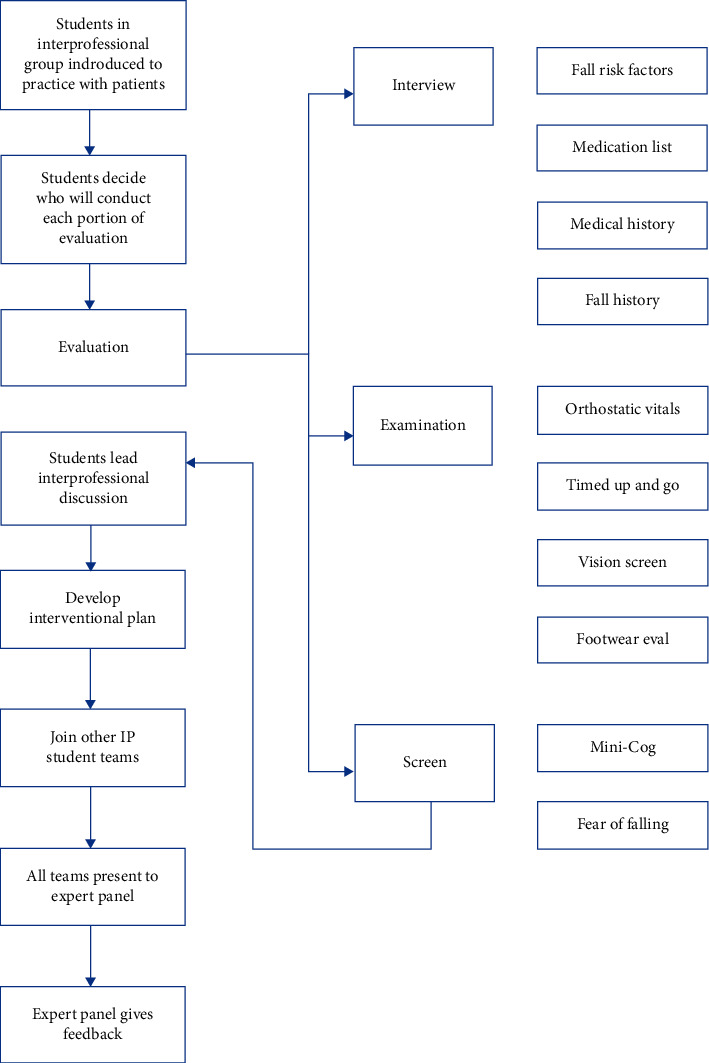
Standardized patient session flow.

**Figure 2:**
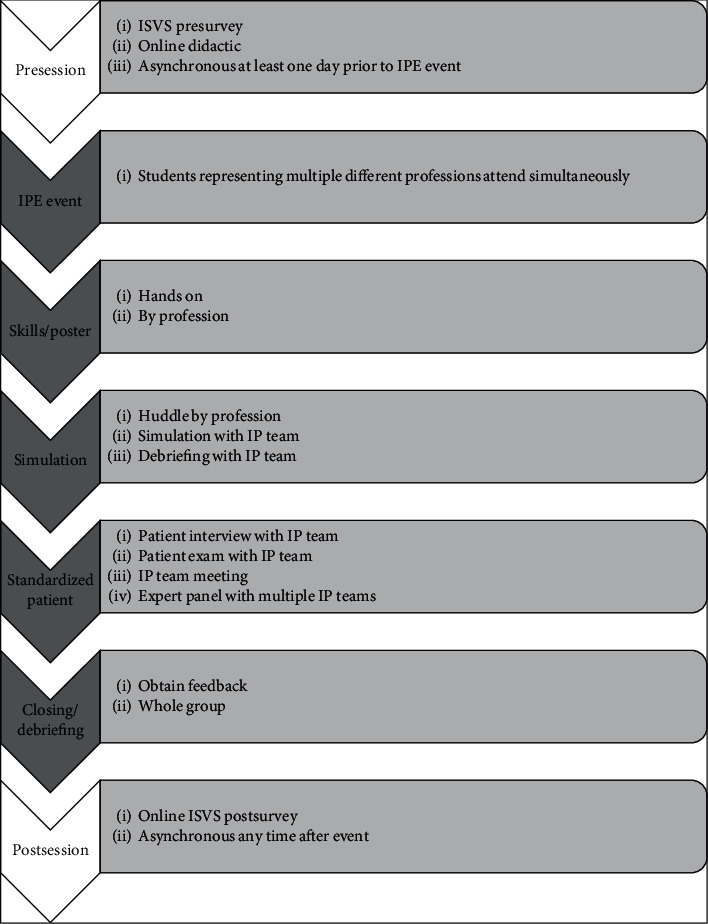
Current educational program.

The PDSA rapid-cycle improvement allowed for continued adjustments and created a modular curriculum that could be modified for the needs and abilities of the students, volunteers, facilitators, and teachers at each session. Every portion of the pilot was reviewed and improved (Table 3 and Figures 1 and 2). The online didactic was felt to be too long and portions were overly detailed for undergraduate and preprofessional students. The graduate students needed more information. As a result of the varying end-user needs, three different didactics were developed. All students were required to view a TeamSTEPPS® didactic and an overview of a geriatric fall prevention didactic [28]. Medical and advanced practice students (NP/PA) along with pharmacy students had an additional didactic focused on advanced testing and interventions. The didactics were self-paced and have optional narration.

The in-person educational session continued to have a poster portion, an IP team simulation session, and a debriefing session as in the pilot (Figure 2). The poster session had been intended to be self-directed, but based on feedback it was made more formal and included additional skills. Students rotated through the poster/skills stations by profession so the facilitator could tailor the instruction and education to the learner needs. Stations included continence, orthostatic hypotension, mobility/function, depression, nutrition, environmental, cognition, TeamSTEPPS®, and pharmacy. The skills that the students practiced were blood pressure measurement, timed up and go, Romberg, footwear assessment, PHQ-9 (Patient Health Questionnaire) depression screen, BMI calculations, home safety checklist for fall risk, Mini Cog, TeamSTEPPS® beat the clock game, and medication assessment for fall risk [28, 32–34]. The TeamSTEPPS® portions allowed the students to practice effective communication [28].

The IP team simulation was done in two parts. First, there was a huddle that was done by a profession. Facilitators provided information to the students to use during the second part of the team simulation that was specific to what they would have obtained by interviewing the case patient. Cases were based on actual FRRC patients. Facilitators gave specific findings that the students would then present to the other students during the team meeting, to simulate the information each profession would contribute to a meeting. Students were not given information that would have been obtained from another profession and only learned the additional information when the team meeting occurred and the different professions presented their findings. For example, chaplain students would not be given physical exam findings that a physical therapist would obtain but learned the findings when the team presented. In turn, the physical therapy students would not learn of the grief since the death of the spouse until the chaplain students presented their findings. Students were then divided into interprofessional teams with 8–10 members. The makeup of the teams depended on types of students participating in the IPE event, with effort made to not have more than 2-3 students of one discipline on a team. The students would run an IP meeting based on the case they received in the huddle. Facilitators were there to ask leading questions if the students became stuck or were unsure of how to proceed. The case scenarios and profession-specific details included depended on which professions were attending the session; for example, if chaplain program students were attending the training, facilitators would include chaplain-specific details in the scenario. If chaplain students were not present, but social work students were, the information might be given to the social work students instead to present. Each IPE event was tailored to the types of students participating. If a particular detail was important for the team, but that profession was not represented on that team due to uneven numbers of participants, the facilitator would play the role of that profession for information sharing.

Early in the course of phase 2, it became obvious that the large number of students attending the educational sessions would not be able to be accommodated in the FRRC for their patient care experience. Only students who were on clinical rotation at the sponsoring hospital with one of the FRRC team members participated in the FRRC. This included nurse practitioner, pharmacy, physical therapy, and medical students. Typically, two-three students attended each FRRC team meeting.

We sought to provide students who could not attend the FRRC with an alternative patient care experience. Based on student feedback, educator input, and HRSA needs, a practical session with a live volunteer standardized patient (SP) was added to the IPE session (Table 3 and Figures 1 and 2). Students remained in their IP groups for the session and each group got a different standardized patient. Students decided amongst themselves who would obtain each portion of the assessment. Often, there was more than one discipline present who could obtain different pieces of information; for example, nursing students or paramedic students could obtain a blood pressure. Facilitators were present to ensure patient safety, answer questions, and maintain session timing but otherwise did not participate in the student-led session. Afterwards, students joined other small IP groups to present their patients and recommendations to an expert panel that included the facilitators who were on the FRRC staff and university educators. The expert panel gave feedback on their plans.

### 2.8. Phase 3: Implementation with a Focus on a Modular Curriculum

Multiple educational sessions were planned on a fluid timeline to allow the opportunity for at least 700 students to participate, as this was a goal of the grant. The PDSA model was used in monthly meetings to make adjustments to the curriculum, presentation plan, participants, teachers, setting, posters, simulation case, and timing. Participation in the educational sessions was voluntary for some students and required for other students as part of their rotation, class or clerkship. Participation in the online survey was voluntary for all participants and required consent to complete as that portion was deemed research by the sponsoring IRB.

## 3. Results

### 3.1. Phase 1

The pilot session was completed in November 2015. The session had 83 students from the three sponsoring institutions (hospital system, university, and medical university). Students were from undergraduate nursing, nurse practitioner graduate program, pharmacy, medicine, social work, physical therapy, nutrition, and pastoral care. The response rate to the ISVS survey was very low with very few students answering the individual questions all three times the survey was requested. Of the 23 questions in the ISVS, the response rate for individual questions ranged from 2 to 31 responses.

The investigator generated attitude and knowledge survey showed 96% percent of students responded that the material presented in the simulation increased their understanding of how an interprofessional team functions. Ninety-five percent of students responded that the simulation experience increased their understanding of the benefits of teamwork in the interprofessional care of geriatric patients.

### 3.2. Phase 3

Since the pilot, 719 students have participated in various manifestations of the online didactic plus in-person training sessions. Ten separate educational sessions have been given at three different institutions. Each session was 4 hours long, but sessions were held at a variety of times and days to accommodate student and institution needs including evening and Saturday. Survey data with demographic information was available on 524 participants. Students came from ten different schools and represented thirteen different health care disciplines (Table 1). Of the 719 students, 370 consented to do the postsurvey online, of those completing the survey, 341/368 (92.7%) felt that their educational session helped them to appreciate the benefits of interprofessional teamwork to a great or very great extent.

We received 33 facilitator evaluations from the sessions that followed the pilot. One hundred percent felt that the simulation scenarios were realistic and the live volunteer patient portion was a good experience of interprofessional teamwork (Table 4). Facilitators were also allowed to leave written comments that are summarized in Table 5.

Due to the large number of students participating in the events and the expansion to additional professional students outside of the sponsoring hospitals associated universities, it was not possible to invite them to the FRRC. Only medical students on elective rotation in geriatrics, pharmacy students, and nurse practitioner students previously scheduled to have precepting time with clinical staff participated. Due to low student numbers, separate data were not collected for FRRC experience.

## 4. Discussion

Our experience with this project shows that a large-scale interprofessional educational session can be created with an IP team approach that includes both clinical and nonclinical educators. Students benefited from geriatric specific education as well as practice working with interprofessional teams during the simulations. Geriatric education is particularly suited to simulation as a means to incorporate interprofessional interactions [15]. Students typically report enhanced realism and immediate feedback provided from standardized patients in medical simulation in comparison to more traditional methodologies [35]. This is especially true when students are learning the effects of their decision-making as a collective group in IPE sessions [36].

Educational sessions like ours emphasize the complex nature of caring for older adults so students understand the value of a whole patient approach. The IP student-led standardized patient evaluation and development of an interventional plan was not part of our pilot but became a strength of our program. Having the students simulate an interprofessional team meeting rather than just a patient care scenario was unique to our program and can be used as a model for others. Within one educational session, students were able to practice IP teams twice-once with the simulation and once with the standardized patient. By having expert panels to give feedback to the student groups on the IP plans, students were able to learn from practitioners who actually function regularly on IP teams. Students also benefited from seeing the other student IP teams present their patients to the panel. The educational experience of interviewing the SP mirrored the experience of the IP team from the FRRC giving the students a practical, close-to-reality exercise without compromising patient or student safety. The educational sessions were enhanced by the simultaneous creation of the hospital FRRC and the interprofessional practice the educators received. The facilitators had recent experience with successfully treating vulnerable older adults with a team approach and could bring those real examples to the group discussion.

We believe that our results can be generalizable to other institutions who have a good working relationship between clinical educators, undergraduate instructors, medical school faculty, and community educators. It may be more difficult to find some local programs such as pastoral care training or a state-accredited EMS school but inviting professionals from those disciplines to the planning phases, educational sessions, and feedback panels may fill that gap. Our simultaneous creation of the IPE with the FRRC allowed grant members to work collaboratively and allowed the students to benefit from our IP relationship. We learned each other's strengths and unique clinical perspectives that allowed us to show the students the benefit of working in a team even when one is considered an expert in their field.

Effective communication was not an issue for our FRRC or our development of the IPE session. This may simply have been a result of our having time to meet in person frequently. Although we have obtained short-term success with our IP program, its development was dependent on funding from a grant. We have had support from educators at local universities not originally associated with the grant who have given their time as facilitators and advisors to the educational content, but grant members have done the bulk of the work. Any institution considering an IPE of this magnitude must be willing to dedicate protected time and resources to the faculty. Although it will require less work to continue with the educational sessions than was required to originally develop them, there must still be a coordinating body to ensure the content is up to date and relevant to the learners. There must also be someone to organize the logistics of the IPE sessions.

We had several limitations to our study. Our response rate to our surveys was low. The students could be required by the instructors to attend our session, but responding to the survey was optional. We were also unable to follow up with students after completion of our training. The IOM recent report “Measuring the impact of interprofessional education on collaborative practice and patient outcomes” highlights the need for research supporting IPE [3]. It is difficult to measure the impact of IP education on patient outcomes as the benefits may lapse by years [3]. With a stable IPE program, it may be appropriate in the future to create longitudinal studies following students into their careers to assess their attitudes toward IP teams, their experience with IP teams, and their willingness to engage others outside their specialty in complex patient problems.

## 5. Conclusion

We created a large-scale interprofessional educational session that incorporates asynchronous learning, simulation, and standardized patients. We used a chronic care model to plan our educational session to highlight why complex patient problems need a team approach. We used a Plan-Do-Study-Act rapid-cycle improvement model to modify our educational session and expand the learning opportunities to hundreds of local students from a wide variety of health care disciplines.

## Figures and Tables

**Table 1:** Phase 3 student types.

Student type	Training level	*N*	%
Nursing/nurse practitioner	Undergraduate/graduate	190	36

Physical therapy/exercise science	Undergraduate/graduate	89	17

Medicine (physician)	Graduate	71	14

Occupational therapy	Undergraduate/graduate	47	9

Social work/counseling	Undergraduate/graduate	35	7

EMS	Certification course	29	6

Pharmacy	Graduate	19	4

Nutrition/dietetics	Undergraduate/graduate	16	3

Pastoral care/chaplain	Certification course	11	2

Speech/language	Undergraduate/graduate	9	2

Physician assistant	Graduate	6	1

Organizational leadership	Graduate	1	<1

Allied health/phlebotomy	Undergraduate	1	<1

Total		524	

**Table 2:** Challenges and solutions identified during phase 2.

Challenge	Solution
Pilot sparked interested from additional hospital and university educators	(1) Invited speech therapy, exercise science, occupational therapy, nutrition, chaplaincy, and social work students and instructors

Additional schools, larger numbers of students	(1) Educational sessions held at 3 different universities
	(2) Some sessions held in evening or on weekend

Students at variety of educational levels	(1) All students viewed presession didactics on TeamSTEPPS® and geriatric fall prevention
	(2) Medical, pharmacy, and advanced practice students viewed additional didactic on evaluation and treatment

Students did not like poster session open flow	(1) Facilitators assigned to poster/skill session
	(2) Students rotated by profession, so instruction matched learner needs

Too many students to participate in FRRC^*∗*^	(1) Some pharmacy, medical, and nurse practitioner students participated with clinic preceptors
	(2) SP^†^ added to educational session
	(3) Students ran IP^‡^ session after SP evaluation

Students required feedback on SP	(1) Students presented SP IP results to expert panel
	(2) Panel consisted of FRRC clinical and university educators

^*∗*^Fall Risk Reduction Clinic. ^†^Standardized patient. ^‡^Interprofessional.

**Table 3:** Current educational session.

Session	Type	Facilitator role	Student pairing
Presession	Online didactic, ISVS presurvey		Individual

Introduction	Overview	Speaker	Whole group

Skills/posters	Hands on	Demonstration/instructional	By profession

Simulation	Huddle	Provide simulation case information	By profession
	Simulation	Guide simulated IP team patient meeting	Small IP groups
	Debriefing	Provide feedback/hear concerns	Small IP groups

Standardized patient (SP)	Patient interview	Ensure patient safety/help students with organization	Small IP groups
	Patient exam	Ensure patient safety/observe skills	Small IP groups
	IP team meeting	Observe	Small IP groups
	Expert panel	Give feedback on student IP plan and additional interventions	Multiple IP groups

Closing/debriefing		Obtain feedback, closing remarks	Whole group

Postsession	Online ISVS postsurvey		Individual

IP: interprofessional; ISVS: Interprofessional Socialization and Valuing Scale.

**Table 4:** Faculty facilitator evaluation (*n* = 33).

Item	Strongly disagree, *N* (%)	Disagree, *N* (%)	Neutral, *N* (%)	Agree, *N* (%)	Strongly agree, *N* (%)	Mean (SD)
The education event was well organized and transitioned well	0	2 (6.1)	1 (3.0)	15 (45.5)	15 (45.5)	4.30 (0.8)

The poster session was organized and valuable to the students	0	0	4 (12.1)	10 (30.3)	19 (57.6)	4.45 (0.7)

The professional huddles helped students understand their roles in the simulation	0	0	4 (12.1)	11 (35.5)	16 (51.6)	4.39 (0.7)

The students were engaged in the simulation scenario	0	0	1 (3.0)	21 (63.6)	11 (33.3)	4.30 (0.5)

The simulation scenario was realistic	0	0	0	15 (45.5)	18 (54.6)	4.54 (0.5)

Students were able to identify patient problems and propose interventions	0	0	3 (9.1)	15 (45.5)	15 (45.5)	4.36 (0.7)

The debriefing sessions provided valuable feedback for students	0	0	2 (6.3)	15 (46.9)	15 (46.9)	4.41 (0.6)

The session with the volunteer provided students with a good experience of interprofessional teamwork	0	0	0	13 (39.4)	20 (60.6)	4.61 (0.5)

This was a valuable learning experience for the students	0	0	0	16 (48.5)	17 (51.5)	4.52 (0.5)

Students worked as a team to develop a plan of care	0	0	5 (15.2)	14 (42.4)	14 (42.4)	4.27 (0.7)

As facilitator, I was able to keep the discussion on track to meet the objectives of the simulation	0	0	3 (9.7)	19 (61.3)	9 (29.0)	4.19 (0.6)

**Table 5:** Facilitator feedback.

Question (*n*)	Comment (*n*)
The most effective part of the simulation experience was (24)	The volunteer patient assessment and care planning (14)
	All disciplines participating (2)
	Working as a team to develop the care plan (3)
	Developing the care plan (1)
	The huddle (1)
	The simulation (2)
	All of it (1)

The least effective part of the simulation experience was (18)	Not having all professions present (3)
	Nothing (3)
	Rooms too crowded (2)
	Sometimes difficult to engage students (2)
	Expert panel hard to hear (2)
	Asking about medications (1)
	The debrief (2)
	Poster time too short (1)
	Presenting care plan to expert panel (1)
	Simulated patient (1)

Recommendations for improving this session for the future (21)	Larger room (3)
	Decrease time (3)
	Engage students more (2)
	None (2)
	More time to give feedback to students (1)
	Have all professions present (1)
	Better coordination of facilitators prior to the event (1)
	Give all students all assessment information (1)
	Restructure debriefs (1)
	More transition time (1)
	Better organization of handouts (1)
	Consistency in case (1)
	Provide large and small blood pressure cuffs (1)
	More time (1)
	Give students the volunteer patient assessments early (1)

Other comments (16)	Great experience for students and faculty (2)
	Well thought out (1)
	Organized (1)
	Didactics were excellent (1)
	Provide more ill volunteer patients (1)
	Students very engaged in the simulation (1)
	Great group, interactive, good problem solvers (1)
	All excellent (1)
	Sparked new ideas for my teaching (1)
	All information should be given to all students (1)
	Unsure of posters, student engagement variable (1)
	Nurses need to speak up more (1)
	More balance academically (1)
	More social work presence (1)
	Decrease time for the volunteer patient evaluation (1)

## Data Availability

The survey data used to support the findings of this study are included within the article, and any additional data used to support the findings of this study are available from the corresponding author upon request.
